# Mouse model of pulmonary cavitary tuberculosis and expression of matrix metalloproteinase-9

**DOI:** 10.1242/dmm.025643

**Published:** 2016-07-01

**Authors:** Alvaro A. Ordonez, Rokeya Tasneen, Supriya Pokkali, Ziyue Xu, Paul J. Converse, Mariah H. Klunk, Daniel J. Mollura, Eric L. Nuermberger, Sanjay K. Jain

**Affiliations:** 1 Center for Infection and Inflammation Imaging Research, Johns Hopkins University School of Medicine, Baltimore, MD 21287, USA; 2 Center for Tuberculosis Research, Johns Hopkins University School of Medicine, Baltimore, MD 21287, USA; 3 Department of Pediatrics, Johns Hopkins University School of Medicine, Baltimore, MD 21287, USA; 4 Department of Medicine, Johns Hopkins University School of Medicine, Baltimore, MD 21287, USA; 5 Center for Infectious Disease Imaging, National Institutes of Health, Bethesda, MD 20892, USA

**Keywords:** Tuberculosis, Cavity, Computed tomography, Matrix metalloproteinases, Mice

## Abstract

Cavitation is a key pathological feature of human tuberculosis (TB), and is a well-recognized risk factor for transmission of infection, relapse after treatment and the emergence of drug resistance. Despite intense interest in the mechanisms underlying cavitation and its negative impact on treatment outcomes, there has been limited study of this phenomenon, owing in large part to the limitations of existing animal models. Although cavitation does not occur in conventional mouse strains after infection with *Mycobacterium tuberculosis*, cavitary lung lesions have occasionally been observed in C3HeB/FeJ mice. However, to date, there has been no demonstration that cavitation can be produced consistently enough to support C3HeB/FeJ mice as a new and useful model of cavitary TB. We utilized serial computed tomography (CT) imaging to detect pulmonary cavitation in C3HeB/FeJ mice after aerosol infection with *M. tuberculosis*. Post-mortem analyses were performed to characterize lung lesions and to localize matrix metalloproteinases (MMPs) previously implicated in cavitary TB *in situ*. A total of 47-61% of infected mice developed cavities during primary disease or relapse after non-curative treatments. Key pathological features of human TB, including simultaneous presence of multiple pathologies, were noted in lung tissues. Optical imaging demonstrated increased MMP activity in TB lesions and MMP-9 was significantly expressed in cavitary lesions. Tissue MMP-9 activity could be abrogated by specific inhibitors. *In situ*, three-dimensional analyses of cavitary lesions demonstrated that 22.06% of CD11b+ signal colocalized with MMP-9. C3HeB/FeJ mice represent a reliable, economical and tractable model of cavitary TB, with key similarities to human TB. This model should provide an excellent tool to better understand the pathogenesis of cavitation and its effects on TB treatments.

## Abstract (precis)

**Summary:** We demonstrate for the first time a murine model that consistently produces pulmonary cavitary TB and provides a tractable and economical new tool for better understanding of cavitation.

## INTRODUCTION

Cavitation of pulmonary lesions is a defining event in individuals with tuberculosis (TB), with negative implications for the individual and for society (Benator et al., 2002; Blumberg et al., 2003; Dorman et al., 2009). For the individual, cavitary TB is associated with poor treatment outcomes, including delayed sputum culture conversion, relapse after treatment and development of drug resistance. For society, cavitation greatly increases the risk of person-to-person transmission. For these reasons, more effective means to treat and prevent cavitary TB are crucial for efforts to shorten the duration of TB treatments, limit the development of drug resistance and reduce transmission. Such efforts would be aided immensely by better understanding of the processes leading to cavitation, and the factors driving reduced treatment responses in patients with cavitary TB.

Cavitation is a consequence of the distinctive caseation necrosis that is the pathological hallmark of human TB. As a result, efforts to develop animal models of cavitary TB have focused on species that exhibit caseation necrosis upon infection with *Mycobacterium tuberculosis* complex, especially rabbits (Converse et al., 1996; Manabe et al., 2003; Nedeltchev et al., 2009; Subbian et al., 2011; Via et al., 2012; Wells and Lurie, 1941; Yamamura, 1958) and non-human primates (NHPs) (Capuano et al., 2003; Via et al., 2013). However, use of these larger animals is often cost prohibitive, and, in the case of rabbits, is challenged by the limited availability of reagents. Furthermore, these models require infection with specific strains (Converse et al., 1996; Subbian et al., 2011; Via et al., 2013; Wells and Lurie, 1941), prior sensitization with heat-killed bacilli, and/or bronchoscopic infection to create cavitary disease (Nedeltchev et al., 2009; Yamamura, 1958). Even then, cavitation can be an infrequent event (Lin et al., 2013). A reliable murine model of cavitary TB would be much more economical and afford a wide array of reagents for investigation of disease mechanisms.

Mice have been overlooked as models of cavitary TB because commonly used mouse strains do not exhibit caseous pathology after infection with *M. tuberculosis*. However, C3HeB/FeJ mice were discovered to develop caseous lung lesions after *M. tuberculosis* infection (Pan et al., 2005); these lesions are now known to be hypoxic (Driver et al., 2012; Harper et al., 2012) and to undergo calcification similar to human TB (Ordonez et al., 2015a). C3HeB/FeJ mice are not deficient in the activation of Th1 cytokine-producing T-cells or their migration to the lungs, and they are able to control infection with Bacille Calmette–Guerin vaccine strains (Yan et al., 2007). Their susceptibility to *M. tuberculosis* infection is determined primarily at the *sst1* locus, which regulates the macrophage innate immune response to infection with intracellular pathogens (Pan et al., 2005; Pichugin et al., 2009). Macrophages of C3HeB/FeJ mice have a reduced ability to restrain multiplication of *M. tuberculosis* and these cells preferentially undergo cell necrosis rather than apoptosis, which is associated with activation of the type I interferon pathway and an exaggerated host inflammatory response (He et al., 2013; Pan et al., 2005). Mounting evidence that type-I-interferon signaling pathways are upregulated in active TB (Berry et al., 2010), as well as evidence associating TB with polymorphisms in *SP110*, the closest human homolog of the candidate *Ipr1* gene of the mouse *sst1* locus, indicates the likely relevance of this mouse strain (Abhimanyu et al., 2011; He et al., 2013; Tosh et al., 2006). Reports of occasional cavitation in C3HeB/FeJ mice (Driver et al., 2012; Lanoix et al., 2015; Ordonez et al., 2015b) support further investigation of their potential as a cavitary TB model. However, to date, there has been no demonstration that cavitation can be produced consistently enough to support such usage. For this study, we utilized serial computed tomography (CT) imaging to detect pulmonary lesions and cavitation in C3HeB/FeJ mice after aerosol infection with *M. tuberculosis* while evaluating the impact of methods to promote cavity formation. Detailed post-mortem histopathological and immunological analyses were also performed to characterize the TB lesions and to probe for matrix metalloproteinases (MMPs) that are implicated in the process of cavitation (Ong et al., 2014; Salgame, 2011).

## RESULTS

### Incidence of cavitary disease

In a series of experiments, C3HeB/FeJ mice infected with *M. tuberculosis* H37Rv were imaged using CT at predetermined time points (Table 1). In one experiment, 4 of 9 untreated mice imaged at 8 weeks post-infection (wpi) had cavitary lesions. In other experiments, 14 of 31 mice and 10 of another 20 mice had cavities at 10 and 14 wpi, respectively. Thus, the combined proportion of untreated mice developing cavities by 8-14 weeks post-infection was 47% (28 of 60). Because cavitary disease is classically associated with reactivation TB, we also evaluated the incidence of cavitation during relapse after non-curative drug treatment. At 6 weeks after infection with *M. tuberculosis* H37Rv, mice received standard TB treatment [8 weeks of rifampin, isoniazid and pyrazinamide (RHZ), then another 4 weeks of RH]. CT was performed 4 weeks after completion of treatment (22 wpi), at which time 11 of 18 (61%) mice had cavities (Table 1) (Ordonez et al., 2015b). These mice (with cavitation) appeared substantially healthier than those that developed cavities in the context of untreated infection.

**Table 1.**
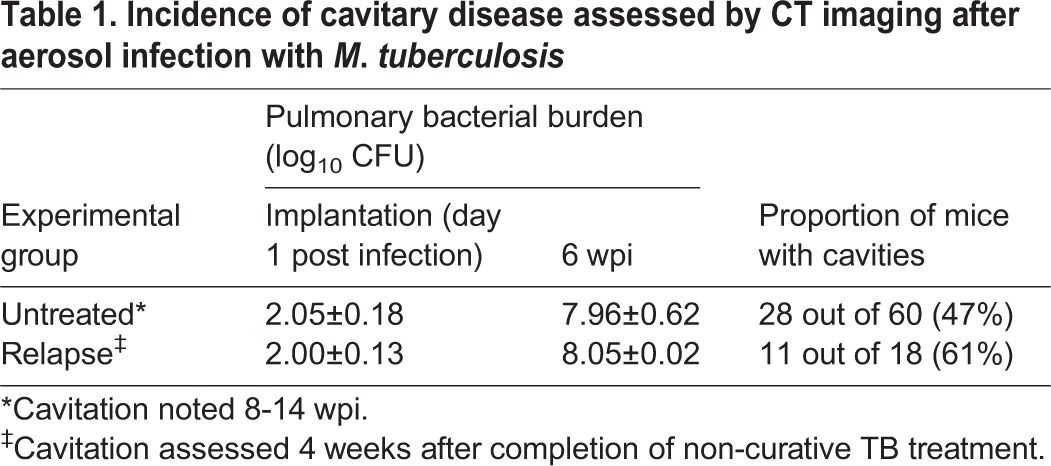
**Incidence of cavitary disease assessed by CT imaging after aerosol infection with *M*. *tuberculosis***

Prior studies in rabbits and NHPs induced cavitation by sensitizing with heat-killed bacilli prior to challenge with viable *M. tuberculosis* (Nedeltchev et al., 2009; Yamamura, 1958) or infections with Beijing lineage *M. tuberculosis* (Subbian et al., 2011; Via et al., 2012, 2013). We therefore compared cavitation in sensitized or un-sensitized mice following infection with *M. tuberculosis* HN878. Unexpectedly, sensitization protected against early death and cavitation (Fig. S1). Survival was higher among sensitized mice (median 136 days) compared to un-sensitized mice [median 107 days; log-rank (Mantel-Cox) test *P*<0.01]. One-quarter of un-sensitized mice died within 44 days post-infection (dpi), whereas no sensitized mouse died before 93 dpi. The improved survival among sensitized mice was associated with better immune containment of infection. At 6 wpi, the pulmonary bacterial burden was lower in sensitized [5.97±0.14 log_10_ colony forming units (CFU)] versus un-sensitized (7.77±1.83 log_10_ CFU) mice [*P*=0.14; Mann–Whitney test; two of the four mice in the un-sensitized group had a very high bacterial burden (>9.3 log_10_ CFU)]. In the absence of drug treatment, none of 15 mice in the sensitized group developed cavities versus 2 (40%) of 5 surviving mice in the un-sensitized group by 14 wpi (Fisher's exact test, *P*=0.05). Among mice treated with RHZ for 8 weeks beginning at 6 wpi, cavitation rates during the 24-week post-treatment follow-up period were 13% (2 of 15) in the sensitized group and 53% (8 of 15) in the un-sensitized group (Fisher's exact test, *P*=0.05).

### Cavitary lesions

**Fig. 1.**
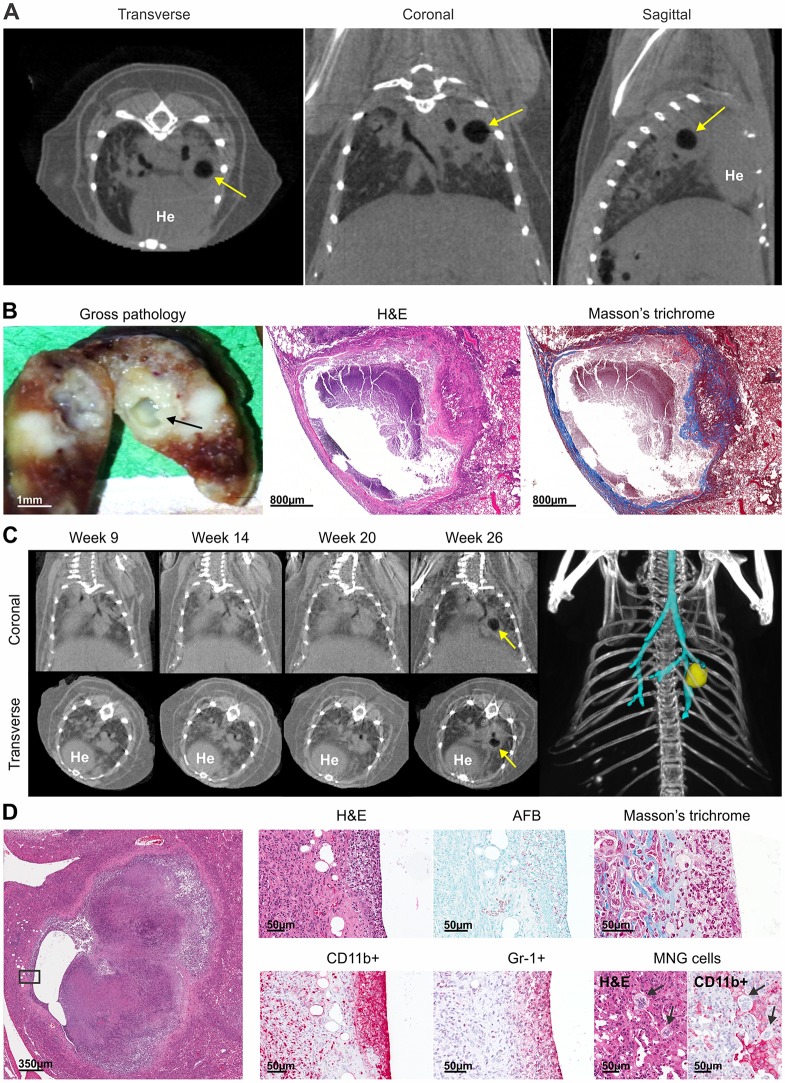
**Cavitary lesions in C3HeB/FeJ mice.** (A) Transverse, coronal and sagittal CT images from a representative *Mycobacterium tuberculosis*-infected (HN878, 29 wpi, after standard TB treatment from 6-14 wpi) mouse demonstrating a thick-walled lung cavity (yellow arrow) are shown. (B) Post-mortem examination of the same animal demonstrated a cavity with residual, partially liquefied caseous material (arrow), a thin cellular rim seen with hematoxylin-eosin (H&E) stain and a fibrotic capsule (Masson's trichrome stain; blue). (C) Serial CT imaging of another representative mouse infected with *M. tuberculosis* HN878 that received standard TB treatment from 6-14 wpi, showing a decrease in the size of an inflammatory mass in the left lung between 9-14 wpi, followed by an increase in the size of the mass and evolution of a cavity (yellow arrows) 12 weeks after discontinuation of treatment (26 wpi). Three-dimensional reconstruction of the skeleton (gray), airways (blue) and cavity (yellow) derived from the CT images is also shown. (D) Low-power view of a representative cavity with necrotic content from another *M. tuberculosis*-infected mouse (H37Rv, 14 wpi) is shown. High-power view of the cavity wall (inset) demonstrates inflammatory cells (H&E) with numerous extracellular and intracellular acid-fast bacilli, revealed with Ziehl-Neelsen stain (AFB), and collagen deposition (Masson's trichrome stain, blue). Immunohistochemistry analysis reveals a high density of CD11b+ (red) and some Gr-1+ (red) cells. Multinucleated giant (MNG) cells, which are CD11b+, can also be observed in the infected tissues. Black arrows, MNG. He, heart.

Pulmonary cavities were clearly visualized on CT imaging of live *M. tuberculosis*-infected animals (Fig. 1A). On post-mortem analysis, cavities were surrounded by a fibrotic capsule and often contained residual, partially liquefied caseum (Fig. 1B). Serial CT imaging demonstrated the development of cavities within enlarging necrotic masses and the close association of cavities with major airways (Fig. 1C). High-power microscopic views demonstrated mixed inflammatory cellular infiltrates lining the cavity wall, numerous extracellular acid-fast bacilli, as well as intracellular bacilli inside neutrophils and foamy macrophages, and a fibrotic rim surrounding the cavity. Immunohistochemistry demonstrated a high density of CD11b+ and Gr-1+ cells, with morphological characteristics of macrophages and neutrophils, respectively. CD11b+ multinucleated giant cells were also observed (Fig. 1D). A similar architecture surrounding the caseous core – consisting of cellular infiltrate and CD11b+ multinucleated giant cells, numerous acid-fast bacilli, and fibrosis – was also noted in necrotic but non-cavitary lesions (Fig. S2).

**Fig. 2.**
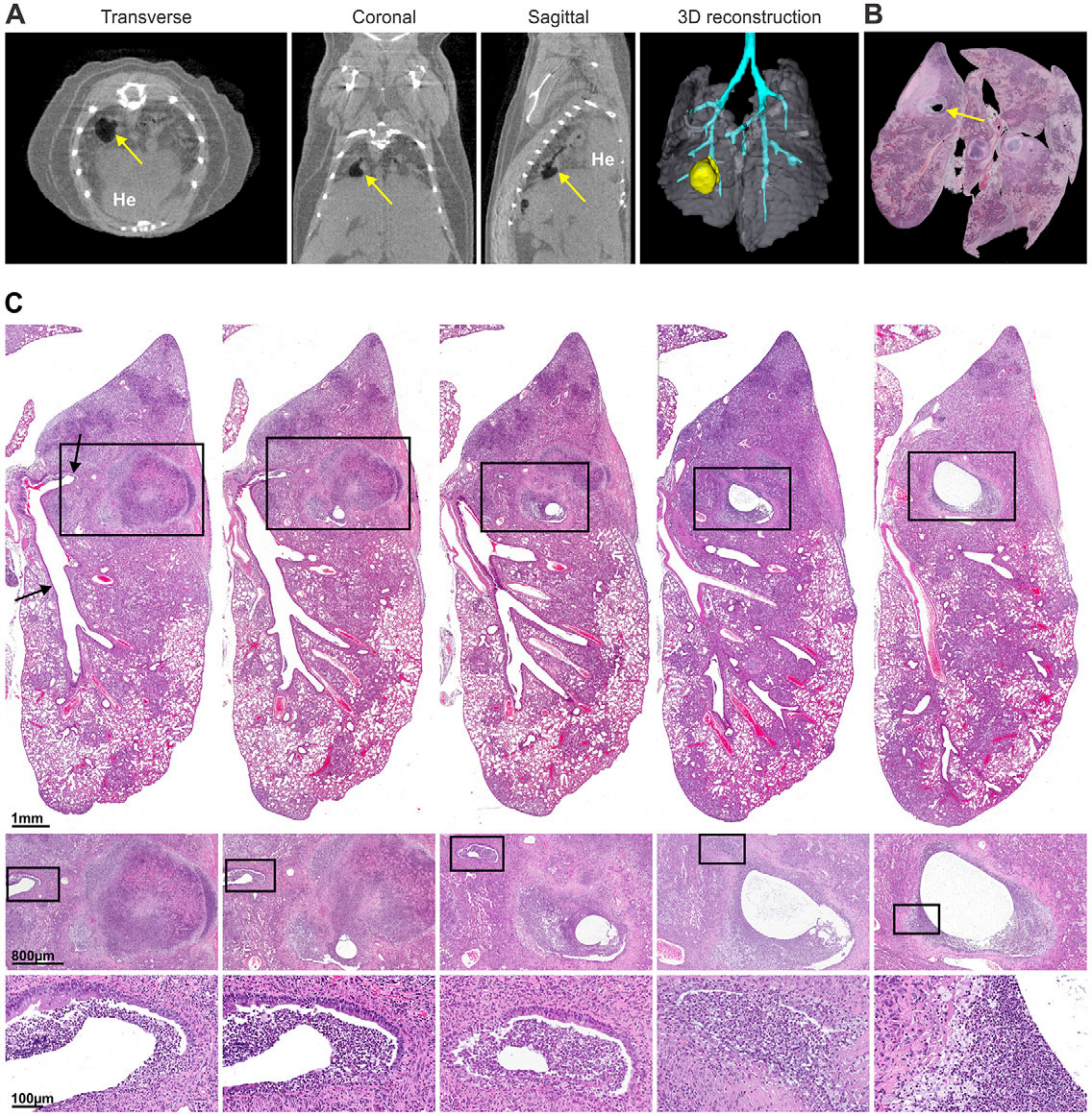
**Communication** **of the cavitary lesion with airways.** (A) Transverse, coronal and sagittal views, and three-dimensional reconstruction derived from CT images from a representative *M.* *tuberculosis*-infected (H37Rv, 8 wpi; without TB treatment) mouse are shown. The airways (blue) directly communicate with the cavity (yellow, arrows). (B) Post-mortem three-dimensional alignment of 98 hematoxylin-eosin (H&E)-stained consecutive histopathological sections of the lung from the same animal shows the cavity (arrow). (C) Consecutive H&E-stained histopathological sections from the same animal demonstrate the communication between a caseating granuloma with cavitation and an adjacent airway. Arrows, airway communicating with the cavity. The (enlarging) lumen of the cavity is seen in the consecutive serial sections, with partially liquefied retained caseum at the periphery. High-power views (insets) of each section demonstrate intraluminal cellular and necrotic debris within the airway. He, heart.

We investigated the relationship between major airways and cavitary lesions (Fig. 2, Movie 1). CT imaging (Fig. 2A) and consecutive post-mortem histopathological sections from the same animal (Fig. 2B,C) demonstrated direct communication between the airway and the partially evacuated cavity. Debris was noted in the airways (Fig. 2C, inset). Another lesion evacuating into the airways is shown in Fig. S3. Cellular and necrotic debris or casts were seen inside the airways, with numerous acid-fast bacilli.

### Lesion heterogeneity and simultaneous presence of multiple lung pathologies

**Fig. 3.**
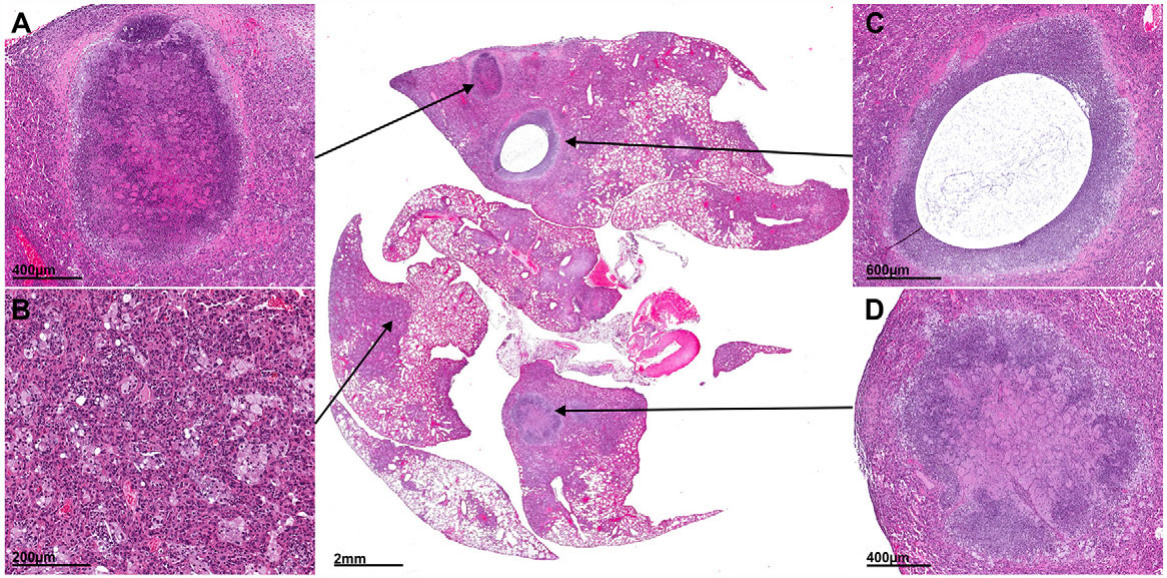
**Lesion heterogeneity.** Histopathological section from the lung of a representative *M*. *tuberculosis*-infected (H37Rv, 14 wpi; without TB treatment) mouse is shown. Higher-power views (insets) demonstrate multiple pathologies – granulomas with central caseation (A,D), pneumonia (B) and cavitation (C) – in different areas of the same lung tissue simultaneously.

Lesions in various stages of development were noted simultaneously during the course of infection (Fig. S4). By 2-4 wpi, the intra-alveolar spaces were occupied by a cellular infiltrate (Fig. S4A) that evolved into organized granulomas with fibrosis and central necrosis (4-6 wpi; Fig. S4B,C). At later stages (8-14 wpi), granulomas became organized, with central caseous necrosis and increasing numbers of extracellular bacilli (Fig. S4D,F). The associated fibrosis progressed from small collagen fibers in the alveolar walls to an organized fibrotic ring surrounding the granuloma. Multiple pathologies – caseous pneumonia, necrotizing granulomas and cavitary lesions – were often seen alongside cellular lesions within the same lung (Fig. 3), as previously described for C3HeB/FeJ mice (Irwin et al., 2015), but with the added feature of cavitation.

### MMP-9 expression

**Fig. 4.**
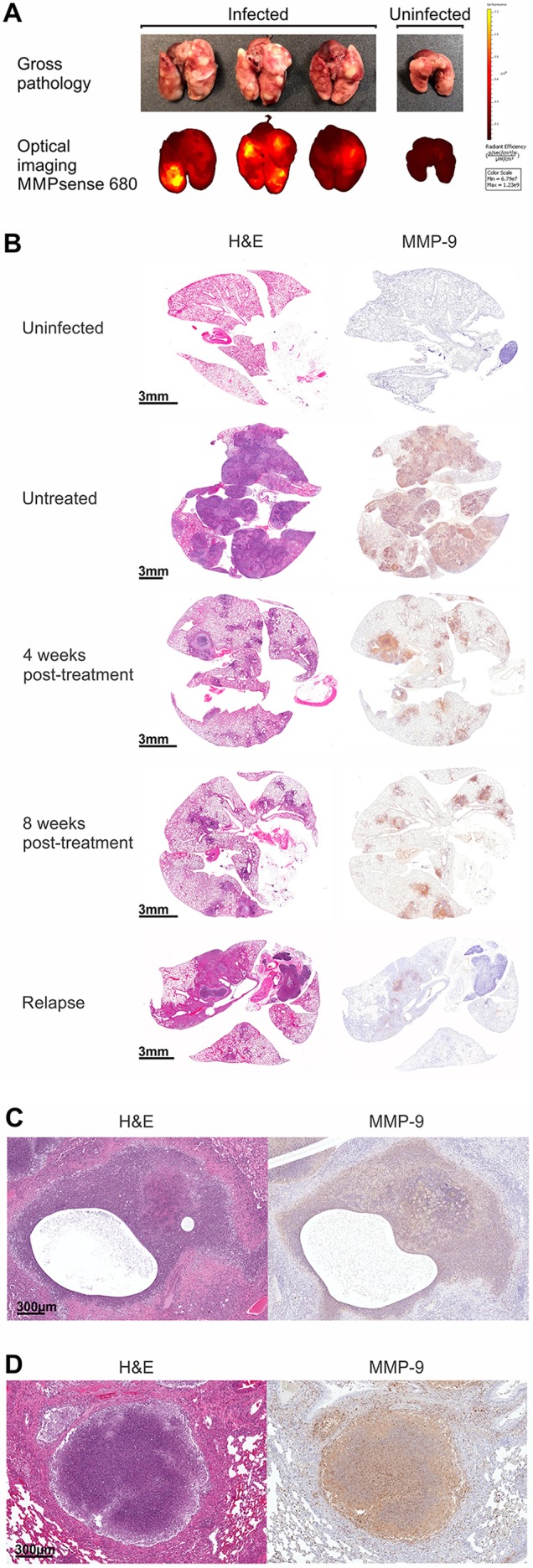
**MMP-9 expression.** (A) Lungs from *M*. *tuberculosis*-infected (H37Rv, 14 wpi, untreated) and age-matched uninfected (control) mice were harvested and imaged in sealed transparent polycarbonate containers using an IVIS Lumina II imaging system 24 h after intravenous injection of MMPSense 680. A greater fluorescent signal was observed in the lung fields of the infected animals, which colocalized with TB lesions (gross pathology). No fluorescence signal was noted in the uninfected animal. (B) MMP-9 immunostaining was performed on lung tissue sections from *M. tuberculosis*-infected (H37Rv, untreated 14 wpi; after 4 and 8 weeks of standard TB treatment and at relapse 12 weeks after completing 8 weeks of treatment) or age-matched uninfected (control) mice. Representative sections from each group are shown. Increased expression of MMP-9 was noted in infected mice at the sites of infection, with a time-dependent decrease during standard TB treatment. Greater MMP-9 expression was also noted in TB lesions at the time of relapse compared to mice at the end of 8 weeks of treatment, whereas no signal was noted in the lungs of uninfected (control) animals. (C) A cavitary lesion from an *M. tuberculosis*-infected mouse (H37Rv, untreated 16 wpi) stained with hematoxylin-eosin (H&E) demonstrated increased MMP-9 expression. (D) A granuloma with central necrosis also demonstrated increased MMP-9 expression.

**Fig. 5.**
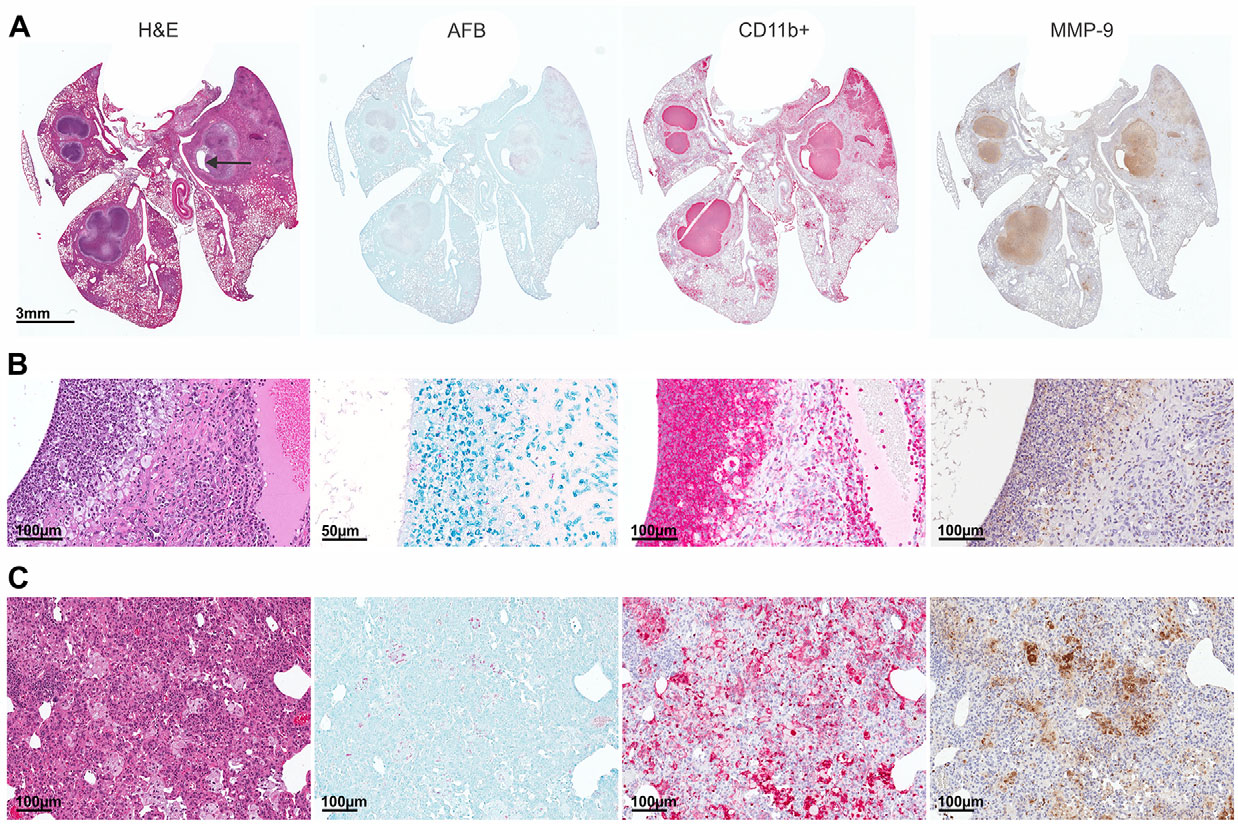
**Association of MMP expression with macrophages.** Hematoxylin-eosin (H&E), Ziehl-Neelsen (AFB), macrophage (CD11b+, red) and MMP-9 (brown) staining was performed on lung tissue sections from a representative *M*. *tuberculosis*-infected (H37Rv, 14 wpi, without TB treatment) mouse with a cavitary lesion (arrow). (A) Expression of MMP-9 was noted at the sites of infection and colocalized with infected areas and the CD11b+ signal. Higher-power views demonstrate CD11b+ and MMP-9 expression in the cellular rim surrounding cavities (B) and in the adjacent pneumonia (C). Numerous bacteria were noted in these lesions.

Destruction of lung extracellular matrix is necessary for the formation of cavities, and MMPs, a family of zinc-dependent proteases, are known to degrade components of the extracellular matrix (Greenlee et al., 2007). MMP activity was qualitatively evaluated in the lungs of *M. tuberculosis*-infected mice with MMPSense 680, an *in vivo* imaging agent activated by key MMPs, including by MMP-9. Fluorescent optical imaging clearly demonstrated increased MMP activity, which colocalized with TB lesions (Fig. 4A). Immunohistochemical analysis revealed increased expression of MMP-9 in the lungs of infected mice, with the signal colocalizing to TB lesions. Time-dependent decreases in MMP expression were observed during standard TB treatment, followed by increased expression in lesions of relapsing mice (Fig. 4B). No signal was noted in the lungs of age-matched uninfected mice. High-power views demonstrated robust MMP-9 expression surrounding cavities and in and around necrotic lesions (Fig. 4C,D). To complement immunohistochemistry, we measured MMP-9 activity in lung homogenates by using gelatin zymography (Fig. S5). MMP-9 activity was noted in lungs from infected mice, but not from uninfected controls. Furthermore, treatment of lung homogenates from *M. tuberculosis*-infected animals with known MMP inhibitors [EDTA (10 mM) and doxycycline (0.25 mM)] (Gendron et al., 1999; Liu et al., 2003), as well as with a highly specific MMP-9 inhibitor, SB-3CT (500 µM) (Gu et al., 2005), abrogated MMP activity. MMP-9 signal also colocalized with CD11b+ cells, which were abundant in the areas surrounding cavities and necrotic lesions (Fig. 5A,B). MMP-9 signal also colocalized with CD11+ cells in pneumonic areas (Fig. 5C). Finally, MMP-9 signal colocalized with the majority of *M. tuberculosis*-infected cells in areas surrounding cavities and pneumonic areas.

### Spatial localization of MMP signal with inflammatory cells

We stained and digitally scanned 36 consecutive sections through a pulmonary cavitary lesion from a representative *M. tuberculosis*-infected mouse (Fig. S6, Movie 2). MMP-9 demonstrated significant colocalization with CD11b+. Quantitative analyses demonstrated that 22.06% of CD11b+ versus 3.75% of Gr-1+ cells colocalized with the MMP-9 signal (Chi-square test with Yates' correction, *P*<0.01).

### MMP-7 expression

Because necrotic TB lesions in this model are hypoxic (Driver et al., 2012; Harper et al., 2012) and MMP-7 is induced by hypoxia (Burke et al., 2003), we also investigated MMP-7 expression. Similar to MMP-9, immunohistochemical analysis revealed increased expression of MMP-7 in TB lesions and time-dependent decreases in MMP-7 expression during standard TB treatment, followed by increased expression in lesions during relapse (Fig. S7). No signal was noted in the lungs of age-matched controls. High-power views also demonstrated MMP-7 expression surrounding cavities and in and around necrotic lesions. Spatial localization demonstrated that 16.74% of CD11b+ versus 2.37% of Gr-1+ cells colocalized with the MMP-7 signal (Chi-square test with Yates' correction, *P*<0.01).

## DISCUSSION

Animal models have key roles in the study of TB pathogenesis and in the development of new therapeutic and preventative measures. However, considering the importance of cavitary pulmonary TB, the development of models representing this pathological feature has been remarkably limited. Our results demonstrate, for the first time, that C3HeB/FeJ mice can serve as a reliable model of cavitary TB to complement models using larger species (Capuano et al., 2003; Converse et al., 1996; Manabe et al., 2003; Nedeltchev et al., 2009; Subbian et al., 2011; Via et al., 2012, 2013; Wells and Lurie, 1941; Yamamura, 1958). In addition to being less economical and less tractable than mice, larger species have other disadvantages. Rabbits are relatively resistant to *M. tuberculosis* infection and have traditionally required infections with *Mycobacterium bovis* (Converse et al., 1996; Wells and Lurie, 1941) or specific strains of *M. tuberculosis* (Subbian et al., 2011), or sensitization with heat-killed bacilli prior to bronchoscopic infection to create cavities (Nedeltchev et al., 2009; Yamamura, 1958). Cavitation occurs in NHPs after *M. tuberculosis* infection (Capuano et al., 2003; Lin et al., 2013; Via et al., 2013), but the frequency of cavitation may not be very high [e.g. 4 of 26 (15%) among bronchoscopically infected cynomolgus macaques] (Lin et al., 2013). Up to 50% (i.e. 3 of 6) marmosets developed cavities after aerosol infection, but only with one of three strains of *M. tuberculosis* complex (Via et al., 2013). Reliance upon *M. bovis* or specific *M. tuberculosis* lineages could limit generalizability to human TB. One example is the intrinsic resistance of *M. bovis* to pyrazinamide – a first-line TB drug that is also a critical component of novel drug regimens under development. The impact of caseous pathology on pyrazinamide's activity and its contribution to combination chemotherapy has recently been elucidated in *M. tuberculosis*-infected C3HeB/FeJ mice (Lanoix et al., 2016a,b). Similarly, sensitization with heat-killed bacilli and bronchoscopic *M. tuberculosis* infection promotes cavitation in rabbits, but also increases the cost and complexity of the model.

We utilized serial CT imaging to rigorously quantify and characterize pulmonary cavitation in C3HeB/FeJ mice after aerosol infection with two *M. tuberculosis* strains. Radiological imaging has been used extensively to assess pulmonary TB (Davis et al., 2009; Kübler et al., 2015; Lin et al., 2013; Ordonez et al., 2015b; Via et al., 2012) and, more recently, as a biomarker of treatment responses in humans (Chen et al., 2014; Salazar-Austin et al., 2015). It is noninvasive and provides rapid, three-dimensional views of the whole organ. It also has the fundamental advantage of longitudinal assessments to monitor disease progression in the same individual, which could also provide new insights into the pathophysiology of disease that are not feasible with conventional methods. We observed pulmonary cavities in 47-61% of C3HeB/FeJ mice, a rate that may be ideal to study the factors associated with cavitation and the impact of cavitation on treatment outcomes. The cavitation rate after *M. tuberculosis* HN878 (East Asian lineage) infection (40%) was similar to that observed with *M. tuberculosis* H37Rv (Euro-American lineage) (47%), although mice infected with HN878 were more likely to require euthanasia prior to the first imaging time point. Further work is needed to determine whether other *M. tuberculosis* strains cause cavitation at similar rates. We also evaluated the effect of sensitization with heat-killed bacilli prior to infection with *M. tuberculosis* HN878 (Beijing subfamily). Unlike prior observations in rabbits (Nedeltchev et al., 2009; Yamamura, 1958), sensitization was protective in C3HeB/FeJ mice, leading to increased survival, lower pulmonary bacterial burdens, and lower rates of cavitation during progressive disease and relapse compared to un-sensitized animals. It is likely that the lower rates of cavitation in sensitized mice was the result of an immunizing effect of the sensitization procedure that restricted initial bacterial growth, as evidenced by the lower lung CFU counts of sensitized mice, and reduced the number and size of caseating lesions in favor of cellular granulomas that do not caseate and thus do not cavitate (Irwin et al., 2015). BCG vaccination has been associated with reduced bacillary burden at the early stages of infection in *M. tuberculosis*-infected C3HeB/FeJ mice, with delayed formation of necrotic granulomas (Yan et al., 2007). Therefore, it is likely that immunization in this model may delay, but not prevent, the formation of cavitary granulomas, as well. Additional longitudinal studies using live imaging are required to clarify the impact of immunization on the progression trajectories of individual granulomas. Similar responses that limit *M. tuberculosis* burden at the site of primary infection (mostly lungs) are noted in primary disease in children who were administered BCG (WHO, 2011). Rabbit models of cavitary TB in which sensitization promotes cavitation employ higher infectious doses (i.e. 10^3^-10^4^ CFU) that are delivered in a liquid vehicle to a small area of the lung by bronchoscope (Kübler et al., 2015; Nedeltchev et al., 2009). In contrast, our study of the effect of sensitization in C3HeB/FeJ mice was limited to an infectious dose of <10^2^ CFU distributed via aerosol throughout the lung. Because higher infectious doses might circumvent the initial growth-restrictive effect of prior sensitization, it would be premature to conclude that sensitization does not promote cavitation in C3HeB/FeJ mice until higher challenge doses closer to those used in sensitized rabbit models are studied.

Given that cavities are typically observed in adult (reactivation) TB (Miller and Miller, 1993), we evaluated the incidence of cavitation after non-curative combination chemotherapy, and observed that 11 of 18 (61%) mice developed cavities. Importantly, mice developing cavities during relapse appeared to be substantially healthier than those that developed cavities in the context of untreated infection (e.g. less activity, ruffled fur, weight loss). Mice that were held for a further 4-8 weeks after observing a cavity via imaging were seen to remain healthy, without clinical worsening, suggesting the potential for a chronic, cavitary TB model.

*M. tuberculosis* is notable for complex interactions with the host. Multiple lesion types can occur simultaneously in the lungs of individuals with active disease, including pneumonic lesions, caseating and non-caseating granulomas, and cavitary lesions (Hunter, 2011; Lenaerts et al., 2015; Nuermberger, 2008). Softening of caseous lesions leading to liquefaction and cavitation is associated with explosive growth of tubercle bacilli, resulting in very high bacillary burdens (10^7^-10^9^ CFU) (Canetti, 1965; Kaplan et al., 2003), and with a significantly higher sputum bacillary load (Palaci et al., 2007). If these lesions or their associated host microenvironments are important for the pathogenesis of human TB or the response to TB drug therapy, it may be essential to experiment in animals that can reliably replicate these pathological states. The cavities observed in C3HeB/FeJ mice share features of human TB – air-filled lesions with caseous debris in various stages of liquefaction and mixed inflammatory cells lining the cavity wall, and encircling fibrotic rims. The cellular infiltrate lining the cavity wall is rich in macrophages (C11b+) and neutrophils (Gr-1+). Multinucleated giant cells and cholesterol crystals, characteristic of human TB lesions (Hunter, 2011), are also observed. CT imaging revealed cavities communicating with major airways containing intraluminal cellular and necrotic debris with numerous intra- and extra-cellular acid-fast bacilli, indicating that the cavities in this model developed when liquefied caseum was evacuated from these lesions through the airways. Serial imaging and necropsies revealed cavities within the necrotic center of organized caseous lesions situated within larger zones of caseous pneumonia, findings described in other models of cavitary TB (Kübler et al., 2015; Via et al., 2013), and in affected humans (Canetti, 1955). Moreover, lesions in various stages of development with multiple pathologies – pneumonia, necrosis and cavitation – were often seen within the lung tissue of the same mouse, suggesting lesion-specific progression of pathology as described in humans and NHPs (Canetti, 1955; Kaushal et al., 2012). However, the development of necrotic granulomas from progressive and coalescing pneumonic lesions cannot be entirely ruled out. The presence of multiple different pulmonary lesions in C3HeB/FeJ mice was described recently (Irwin et al., 2015), but cavitation was a rare event under the conditions evaluated. Because several TB drugs with differing physicochemical properties have been shown to partition differently into these lesions in a manner similar to observations in humans and/or rabbits (DeMarco et al., 2015; Irwin et al., 2016; Kjellsson et al., 2012; Lanoix et al., 2016b; Prideaux et al., 2015; Weinstein et al., 2012), and because the activity of some drugs might be modulated by conditions within large caseous lesions (Lanoix et al., 2015, 2016a), the more tractable and economical C3HeB/FeJ mouse model that exhibits these multiple lesion types provides an important new tool for TB drug development research.

Destruction of lung extracellular matrix is a prerequisite for cavity formation. MMPs, a family of zinc-dependent proteases, are known to degrade several components of the extracellular matrix (Greenlee et al., 2007). The role of MMPs in TB is well summarized in recent reviews (Ong et al., 2014; Salgame, 2011) and multiple MMPs have been associated with TB pathogenesis. In particular, previous studies have implicated MMP-1 (collagenase), MMP-9 (gelatinase) and MMP-7 (matrilysin) in active TB and cavitation (Singh et al., 2014). We qualitatively assessed MMP activity using optical imaging with MMPSense 680, an *in vivo* imaging agent activated by key MMPs, including MMP-9. Optical imaging clearly demonstrated MMP activity, which colocalized with TB lesions seen on gross pathology. Although wild-type mice do not express an ortholog of MMP-1 (Elkington et al., 2011), they do express MMP-7 and MMP-9. The absence of an MMP-1 homolog in mice indicates that this MMP is not essential for cavity formation in this model. Therefore, other collagenases (MMP-8, MMP-13) may play a role in degrading the fibrillary collagen of the lung extracellular matrix in this mouse model. MMP-9 is known to degrade primarily collagen type IV present in the basement membrane of alveoli (Gioia et al., 2009). Immunohistochemistry demonstrated increased expression of MMP-9 surrounding cavitary lesions as well as in and around necrotic lesions, which decreased with TB treatment. This was corroborated by robust MMP-9 activity in infected lung tissues, which was abrogated by known MMP inhibitors (EDTA and doxycycline) (Gendron et al., 1999; Liu et al., 2003), as well as by a highly specific MMP-9 inhibitor, SB-3CT (Gu et al., 2005). These and other MMP-9 inhibitors warrant evaluation as inhibitors of cavitation and lung destruction during TB infection. We and others have previously demonstrated that necrotic TB lesions in C3HeB/FeJ mice are hypoxic (Driver et al., 2012; Harper et al., 2012), and MMP-7 is induced by hypoxia (Burke et al., 2003). Therefore, we also investigated MMP-7 in this model, which was similarly expressed surrounding cavitary and in and around necrotic lesions. Other MMPs (e.g. MMP-8) (Sathyamoorthy et al., 2015) and proteases such as cathepsins (Converse et al., 1996; Kübler et al., 2016, 2015) may also have significant roles, and their evaluation will be the focus of future studies. Our results indicate that C3HeB/FeJ mice could also be useful to develop novel host-directed TB treatments, especially those targeting MMPs.

Given the well-established role of macrophages and growing evidence that neutrophils are also key mediators of inflammation in necrotic and cavitary TB lesions (Berry et al., 2010; Dorhoi and Kaufmann, 2016; Eum et al., 2010; Marzo et al., 2014), we evaluated the spatial location of MMPs and their relationship to macrophages and neutrophils *in situ* in three dimensions. Because MMPs are secreted extracellularly, they are not easily amenable to flow-cytometric analyses. Therefore, we immunostained and digitally scanned 36 consecutive sections through a pulmonary cavitary lesion from a representative *M. tuberculosis*-infected mouse and re-constructed a three-dimensional stack with multiple channels. We analyzed high-resolution images from several consecutive sections across macroscopic dimensions to control for sampling bias. MMP-9 and MMP-7 demonstrated significant colocalization with CD11b+ cells (Chi-square *P*<0.01), suggesting that macrophages are an important source of MMP-7 and MMP-9 in this model, and consistent with published literature (Volkman et al., 2010; Volpe et al., 2006).

In summary, we have demonstrated that C3HeB/FeJ mice reliably develop cavitary pulmonary TB after aerosol infection with *M. tuberculosis*. Serial CT imaging could non-invasively detect cavitary lesions *in vivo* and their communication with major airways. Post-mortem analyses demonstrated that pulmonary lesions in C3HeB/FeJ mice share several key pathological features of human TB, including the presence of multinucleated giant cells, and the simultaneous presence of multiple pathologies – granulomatous pneumonia, necrosis and cavitation – in the same animal. MMP-9 is significantly expressed in cavitary and necrotic lesions, and predominantly associated with CD11b+ cells. C3HeB/FeJ mice present an economical and tractable model of cavitary TB, and warrant further development as a new tool for studying the pathogenesis of cavitation and its effects on TB treatment.

## MATERIALS AND METHODS

All protocols were approved by the Johns Hopkins Biosafety, Radiation Safety, and Animal Care and Use Committees.

### Mycobacterial strains

*M. tuberculosis* H37Rv and HN878 were used as frozen stocks prepared from a log-phase culture in Middlebrook 7H9 broth after mouse passage and were diluted in phosphate buffered saline (PBS) before infection.

### Animal infection and treatments

Four- to six-week-old female C3HeB/FeJ mice (Jackson Laboratories, Bar Harbor, ME) were aerosol-infected with frozen stocks of *M. tuberculosis* using the Middlebrook Inhalation Exposure System (Glas-Col, Terre Haute, IN) (Harper et al., 2012). At least 3 mice were sacrificed the day after infection and at subsequent pre-determined time points to determine CFU counts in the lungs. Some cohorts received rifampin (10 mg/kg body weight), isoniazid (10 mg/kg) and pyrazinamide (150 mg/kg) (RHZ) once daily, by gavage, 5 days per week for 8-12 weeks, beginning 6 wpi, and followed after completing treatment to detect cavitation during relapse. To assess the effect of prior sensitization with mycobacterial antigens on cavity formation, some cohorts received 5 subcutaneous injections of approximately 7 log_10_ CFU of heat-killed *M. tuberculosis* HN878 in incomplete Freund's adjuvant twice weekly beginning 45 days before aerosol infection with the same strain (Nedeltchev et al., 2009).

### Imaging

Live *M. tuberculosis*-infected mice were serially imaged within a sealed biocontainment device (Minerve, Esternay, France) modified in-house to comply with biosafety level-3 (BSL-3) containment (Davis et al., 2009; Ordonez et al., 2015b; Weinstein et al., 2012). Each mouse was imaged using the NanoSPECT/CT small animal imager (Bioscan, Washington, DC). Images were reconstructed and visualized using VivoQuant 2.50 (inviCRO, Boston, MA) or Amira 5.2.1 (FEI, Hillsboro, OR). A cavity was defined as a macroscopic region of air (density<−900 Hounsfield units) within diseased lung parenchyma.

### *Ex vivo* optical imaging

Lungs from *M. tuberculosis*-infected (14 wpi) and age-matched uninfected (control) mice were harvested and imaged in sealed transparent polycarbonate containers using an IVIS Lumina II imaging system (PerkinElmer, Waltham, MA) 24 h after intravenous injection of MMPSense^®^ 680 Fluorescence Imaging Agent (PerkinElmer), an *in vivo* imaging agent activated primarily by MMP-2, MMP-3, MMP-9 and MMP-13 (Jager et al., 2016). The data were analyzed using Living Image software (PerkinElmer).

### Histopathology

Lungs were harvested after systemic perfusion with PBS under deep anesthesia, fixed in 4% paraformaldehyde and sectioned to 5-μm thickness. Hematoxylin-eosin, acid-fast, Masson's Trichrome, reticulin and Picrosirius Red staining was performed following standard procedures (Ordonez et al., 2015b). A cavity was defined by visualization of an air-filled lesion on gross and histopathological assessment.

### Immunohistochemistry

Paraffin-embedded sections were rehydrated in graded alcohols, steamed in citrate buffer at pH 6 and probed at room temperature for 2 h for the following: macrophages [rat monoclonal (M1/70) to CD11b+; 1:500; Abcam], granulocytes [rat monoclonal (NIMP-R14) to Gr-1+; 1:500; Novusbio], and MMP-7 and MMP-9 (rabbit polyclonal; 1:250; Abcam). Both macrophages and granulocytes were then processed using VECTASTAIN AP-Red, (Vector Laboratories, Burlingame, CA) whereas MMPs were processed with a polymer-HRP kit (BioGenex, San Ramon, CA) with diaminobenzidine development and Mayer hematoxylin counterstaining. Lungs from untreated animals, both uninfected and infected, without primary antibody served as negative controls. Slides were scanned using an Apeiro digital scanner (Leica, Buffalo Grove, IL).

### Gelatin zymography

MMP-9 activity was analyzed in frozen lung tissue homogenates. The samples were thawed and incubated in Triton X-100 to release MMPs and run on a 7.5% SDS-polyacrylamide gel electrophoresis with a gelatin substrate (Snoek-van Beurden and Von den Hoff, 2005). Recombinant human MMPs (rhMMP-9 and rhMMP-2; 10 ng/ml) were loaded as controls. Incubation of lung homogenates from *M. tuberculosis*-infected animals with inhibitors of MMP activity was also performed.

### *In situ* spatial localization of MMP signal with inflammatory cells

We analyzed high-resolution data from several consecutive sections across macroscopic data to control for sampling bias. Thirty-six consecutive sections (each 5 µm) through a pulmonary cavity from a representative *M. tuberculosis*-infected mouse were immunostained, digitally scanned at high resolution (Aperio Digital Pathology Slide Scanner, Leica Biosystems, Buffalo Grove, IL) and reconstructed in three dimensions. Each section was manually reviewed for staining characteristics. To analyze the colocalization of CD11b+ and Gr-1+ with MMPs, sequential sections were spatially registered. Immunostains were first converted to luminance, and an affine registration algorithm was utilized based on mutual information (Matlab, MathWorks, Natick, MA). Images were visualized using Amira 5.2.1 (FEI, Hillsboro, OR). A minimum of 300,000 voxels (three-dimensional pixels) were counted for each channel.

### Statistical analysis

Survival was compared using the log-rank (Mantel–Cox) test. Lung CFU counts were log-transformed and expressed as mean and standard deviation (s.d.). Group means were compared using the Mann–Whitney test. Proportions were compared using two-tailed Chi-square with Yates' correction or Fisher's exact test. All analyses were performed using Prism 6.0 (GraphPad, San Diego, CA).
